# Reactive Oxygen Species (ROS) and Nucleic Acid Modifications During Seed Dormancy

**DOI:** 10.3390/plants9060679

**Published:** 2020-05-27

**Authors:** Kai Katsuya-Gaviria, Elena Caro, Néstor Carrillo-Barral, Raquel Iglesias-Fernández

**Affiliations:** 1 Centro de Biotecnología y Genómica de Plantas-Severo Ochoa (CBGP, UPM-INIA), Universidad Politécnica de Madrid (UPM) - Instituto Nacional de Investigación y Tecnología Agraria y Alimentaria (INIA), 28223-Pozuelo de Alarcón, Spain; kai.kgaviria@alumnos.upm.es (K.K.-G.); elena.caro@upm.es (E.C.); 2 Departamento de Biotecnología-Biología Vegetal, Escuela Técnica Superior de Ingeniería Agronómica, Alimentaria y de Biosistemas, UPM, 28040-Madrid, Spain; 3 Departamento de Fisiología Vegetal, Facultad de Ciencias, Universidad da Coruña (UdC), 15008-A Coruña, Spain; n.carrillo@udc.es

**Keywords:** after-ripening, DNA methylation, oxidation, RNA stability, seed dormancy, seed vigour, ROS

## Abstract

The seed is the propagule of higher plants and allows its dissemination and the survival of the species. Seed dormancy prevents premature germination under favourable conditions. Dormant seeds are only able to germinate in a narrow range of conditions. During after-ripening (AR), a mechanism of dormancy release, seeds gradually lose dormancy through a period of dry storage. This review is mainly focused on how chemical modifications of mRNA and genomic DNA, such as oxidation and methylation, affect gene expression during late stages of seed development, especially during dormancy. The oxidation of specific nucleotides produced by reactive oxygen species (ROS) alters the stability of the seed stored mRNAs, being finally degraded or translated into non-functional proteins. DNA methylation is a well-known epigenetic mechanism of controlling gene expression. In *Arabidopsis thaliana*, while there is a global increase in CHH-context methylation through embryogenesis, global DNA methylation levels remain stable during seed dormancy, decreasing when germination occurs. The biological significance of nucleic acid oxidation and methylation upon seed development is discussed.

## 1. Introduction: Seed Dormancy

Seeds are the first world crop, and the basic knowledge applied to enhance complex traits such are dormancy, viability, and vigour is essential for food security and crop production. The seed represents the sexual reproductive entity of higher plants and its development is divided into embryogenesis, maturation, and germination. In the final stage of maturation, seeds begin to dehydrate and acquire tolerance to desiccation and eventually can enter into a latency state named primary dormancy [1]. Dormancy is defined as the incapacity of a mature, dry, and viable seed to germinate under favourable conditions. Non-dormant seeds are able to temporarily block their germination if exposed to unfavourable germination conditions upon seed imbibition. This phenomenon is referred to as secondary dormancy [2,3,4]. Pre-harvest sprouting (PHS) occurs when seeds lose dormancy and germinate in the mother plant before the dispersion takes place. PHS is an important problem in cereal production because it reduces crop yield and quality [5]. The time course of seed dormancy comprises induction, maintenance, and release, and it is well-known that the ABA/GA ratio governs these transitions, although other hormones such as auxins are also involved. In the last decades, several genes have been related to the seed dormancy process, such as those involved in ABA and GA signalling and metabolism, and several transcription factors belonging to B3, bZIP, and RING finger families (ABI3, ABI5, FUS3, DESPIERTO) [6,7,8,9,10]. The *Delay Of Germination-1* (*DOG1)* gene, a quantitative trait locus controlling seed dormancy, is essential to stablish this process, but its specific molecular function is still unknown [11]. The depth of dormancy is directly related to DOG1 protein levels, and mutations or decreased expression of DOG1 during maturation generate seeds with reduced dormancy [12]. It has been recently reported that the DOG1 protein forms a complex with a type of phosphatase 2C named ABA HYPERSENSITIVE GERMINATION1 (AHG1), and this complex is critical for seed dormancy maintenance [13,14,15,16,17]. The state of the art regarding DOG1 is discussed in detail in a review included in this issue [18].

Dormancy is usually classified into the physiological, physical, chemical, morphological, and morpho-physiological categories [19]. Physiological dormancy is abundant in the soil seed bank, and its release is mainly governed by the after-ripening (AR) and stratification processes. Stratification occurs when seeds undergo a time-term of cold moisture that breaks dormancy, and after-ripening takes place when a seed gradually loses dormancy through a period of dry storage. Thus, AR determines the dormancy degree and consequently affects the crop performance in the field [20,21,22]. The period of dry storage is influenced by the environmental conditions experienced by the mother plant during seed development [23]. The AR period is also specific for each species and even for distinct accessions, such as occurs in the *Arabidopsis thaliana* Columbia (Col) and Cape Verde Islands (Cvi) ecotypes, where it ranges from one to six months, respectively [24]. In the dry state, the low water content in the seed prevents metabolic activity, raising the question of how dormancy release occurs at the molecular level. AR and no-AR imbibed seeds show specific gene expression patterns in *Arabidopsis thaliana* (hereafter *A. thaliana*) and in our model of study *Sisymbrium officinal*e [20,25,26,27,28,29]. Interestingly, the seed redox state fluctuates during the AR period and the reactive oxygen species (ROS) quantity increases concomitantly with seed development, promoting dormancy release [30,31]. During the *A. thaliana* and *Hordeum vulgare* seed development, mRNAs are progressively accumulated and some of them remain in the dry seed state, where they are specifically translated depending on the environmental conditions [32,33,34]. The molecular mechanisms controlling the stored mRNA longevity and their specific translation during seed imbibition are widely unknown. However, chemical modifications of mRNA, such as the oxidation caused by ROS, have been related to its stability [35]. Other nucleic acid modifications, such as DNA and RNA methylation, have been also described to be critical for *A. thaliana* embryogenesis and seed viability, facts that demonstrate the importance of these chemical marks in seed biology [33,34,36]. These new findings and their relevance to seed biology make it timely to update this topic. Here, we discuss how mRNA and genomic (gDNA) oxidation and methylation control the expression levels of genes/proteins involved in seed dormancy release, with emphasis in the AR process.

## 2. Reactive Oxygen Species (ROS) Promote Dormancy Release and Modify Stability of Nucleic Acids

### 2.1. ROS Affect Dormancy Release

Reactive oxygen species (ROS), such as hydrogen peroxide (H_2_O_2_), superoxide ion (O_2_^•^), and hydroxyl radicals (OH^•^), are normally produced during cell metabolism. ROS accumulate at different stages of seed development and have been correlated with a low degree of dormancy [31,37,38]. When ROS content reaches a threshold, seed ageing takes place. At this point an intensive degradation of nucleic acids, proteins, and phospholipids present at the cell membrane occurs [39]. Likewise, once the ROS threshold has been reached, dormancy is alleviated and the subsequent germination can be initiated [31,40]. The quantity of ROS depends on the balance between the production and scavenging carried out by the antioxidant systems. In dry seeds, the quantity of ROS increases through storage, and this production mainly occurs by non-enzymatic processes, such as lipid peroxidation and the Amadori–Maillard reaction, but also oxidoreductase enzymes such as NADH oxidases could participate [41,42]. In *A. thaliana* mature seeds, a cuticular film in the outer side of the endosperm (next to the seed coat) has been identified, and this structure could limit the permeability of the seed to outer compounds. Interestingly, when cutin biosynthesis is affected, mutant seeds display a low degree of dormancy and a high level of lipid oxidation [43]. The storage of seeds at dry conditions (AR) and elevated partial pressure of oxygen (EPPO) reduce dormancy faster than AR simply does [38]. Moreover, AR and dormancy release due to EPPO share QTLs such as *DOG1*. Since the seed levels of DOG1 are not reduced during the AR or the imbibition, post-translational changes have been proposed to regulate its action [44]. Recently, it has been reported that DOG1 binds to a heme-group. It is argued that the heme-binding site of DOG1 could act as a sensor of stimuli derived from the presence of oxygen, as it is observed in other heme-binding proteins [11,16].

ROS accumulate in seeds upon AR and dormancy release and promote germination in several species, as illustrated by studies in *A. thaliana*, *Pisum sativum*, *Helianthus annuus*, *Hordeum vulgare,* and *Triticum aestivum* [40,45,46,47,48]. In *A. thaliana* and *H. annuus* AR seeds, while ROS are localized close to the radicle tip of the embryonic axis during imbibition, they do not have a particular distribution in dormant embryos [49,50,51]. It is widely known that ROS act as signalling molecules, but H_2_O_2_ seems to be the main responsible for redox signalling, probably because of its stability. However, OH^•^ and O_2_^•^ radicals are also produced and participate in radicle emergence and embryo growth probably by their participation in cell wall modification [52,53,54]. In AR seeds, ROS have been described to be involved in ethylene signalling and to alter the ABA/GA ratio by promoting the expression of *CYP707A* genes involved in ABA degradation and by increasing GA biosynthesis [45,55,56]. This is in agreement with our previous results in *Sisymbrium officinale,* where nitrate modifies the expression of *ABA* and *GA* metabolic genes in AR seeds [28]. In wheat and barley, while AR dry seeds accumulate H_2_O_2_ and singlet oxygen (^1^O_2_), non-enzymatic antioxidant content diminishes (i.e., ascorbate and glutathione [57,58]). Several enzymes that participate in ROS homeostasis have been associated with the germination and AR process, such as NADPH oxidases (respiratory burst oxidase homologues: rboh), Class III peroxidases (CIII Prx) and thioredoxins (Trxo1). AtRbohB is a major producer of O_2_^•^ in germinating *A. thaliana* seeds, and the *AtrbohB* mutants fail to after-ripen and show reduced protein oxidation. Moreover, the inhibition of O_2_^•^ production leads to a delay in *A. thaliana* and *Lepidium sativum* seed germination [41,59,60]. In *Oryza sativa* seeds, the *PHS9* gene encoding a CC-type glutaredoxin has been involved in the regulation of preharvest sprouting (PHS) through the integration of ROS and ABA signalling [61].

### 2.2. Oxidation Modifies Nucleic Acid Stability during Seed Dormancy

During the AR period, the specific oxidation of mRNAs and proteins (carbonylation) has been described in *Helianthus annuus* seeds [30]. The stored mRNAs in the dry seed can undergo mainly two fates: (i) translation to their corresponding proteins during early seed imbibition and (ii) degradation. The specific mRNA turnover is a prerequisite for dormancy breakage [15]. ROS oxidize nucleic acids at different positions affecting their stability. The 8-hydroxyguanosine (8-OHG) is the most frequent oxidative nucleoside in RNA molecules [62]. The oxidized mRNAs could undergo a ribosome-based quality control to be degraded by the no-go decay pathway (NGD) or translated into non-functional proteins [63]. The oxidation of mRNAs causes premature termination of translation or the appearance of errors in the translation process resulting in the subsequent degradation of the proteins [62]. It has been reported that the RNA polymerase can control the incorporation of 8-hydroxyguanosine (8-OHG) into the growing mRNA strand during transcription and this could be a process that regulates mRNA stability [64]. Dry seeds contain long-lived mRNAs, and the oxidation of these mRNAs has been linked to dormancy release and seed ageing [31,35]. In *Helianthus annuus* and *Triticum spp.* AR-seeds, the oxidation of stored mRNAs encoding enzymes involved in nutrient storage (α-amylase/trypsin inhibitors) has been related to dormancy release [30,65,66]). A decrease in the abundance of stored mRNAs associated to dormancy has been observed in *A. thaliana* AR-seeds, such as that encoding the DELLA protein GAI [67].

**Figure 1:**
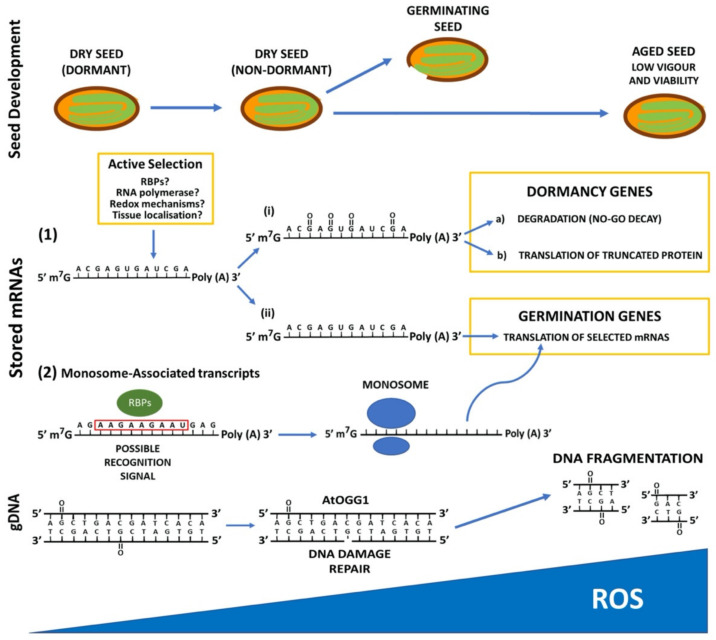
Schematic representation of how reactive oxygen species (ROS) affect seed dormancy and viability by the oxidation of nucleic acids (mRNA and gDNA). Blue circles depict the ribosome.

During maturation, seeds accumulate mRNAs, and some of them are degraded through the AR period. This degradation is caused presumably by oxidation, thus altering their stability. However, some mRNAs are stable and endure until germination. Those mRNAs that are translated during early seed germination are highly specific, and they seem to be selected by environmental conditions [32,35]. The question of how stored long-lived mRNAs remain stable until germination and how they are selected is poorly understood. It has been proposed that RNA binding proteins (RBPs) recruit mRNAs, protecting them during the storage period. These RBPs recognize different types of motifs in the RNA sequence, such as the DEAD-box helicase domain that can activate or repress translation [68]. The RBPs can also bind to the ribosome, thus controlling translation. Moreover, ribosomes can be stored in quiescent states, such as occurs in bacteria, yeast and animals [69]. It has been described that certain characteristics of mRNAs determine its association to ribosomes, such as transcript length, GC content, or the presence of upstream open reading frames (uORFs). It has been argued that the mRNA selection by RBPs lies in the presence of specific motifs in their sequences [70]. In *A. thaliana* dry seeds, stored mRNAs are mainly associated with monosomes that are transcribed during seed maturation and translated upon early germination [22]. Recently, a consensus motif (5′- GAAGAAGAA- 3′) that is significantly overrepresented in 5′-UTR monosome-associated transcripts has been identified, suggesting that it could play a role in the recruitment of specific RNA-binding proteins [70] (Figure 1).

Stored seeds also suffer DNA damage caused by oxidation, which affects seed longevity. The oxidation of the guanine at the C-8 position to produce 7,8-dihydro-8-oxoguanine (8-oxoG) is the most common modification involved in DNA damage [71]. Overexpression of a DNA glycosylase/apurinic/apyrimidinic lyase (AtOGG1), involved in repairing DNA by eliminating 8-oxoG, enhances longevity in *A. thaliana* seeds [72]. ROS also affect DNA methylation by the oxidation of the methyl-5’-cytosine (m^5^C), which is more vulnerable to oxidation [73]. During *Pyrus communis* and *Quercus robur* seed storage, DNA methylation pattern is modified, and this is correlated to the ROS levels in the seed [74,75].

## 3. mRNA and DNA Methylation upon Seed Development

### 3.1. The Seed Epitranscriptome

Non-classical chemical changes, apart from capping and polyadenylation modifications, alter the stability of mRNAs,. More than 160 chemical modifications of mRNAs have been recently reported in *A. thaliana*, as a whole they are considered the epitranscriptome [76]. These alterations include the methylation of the adenosine and cytosine nucleotides: N6-methyladenosine (m^6^A) and 5-methylcytosine (m^5^C) [77]. These mRNA modifications modulate transcript stability, and they have been found in transcripts that are being degraded, suggesting they are involved in mRNA turnover. However, stable mRNAs contain chemical modifications related to the alternative splicing of introns. This fact indicates that these modifications could also regulate the expression of different transcript variants [78]. mRNA chemical modification has been recently described as essential for plant development, and it is considered a new emerging level of controlling gene expression [76]. In *A. thaliana*, the first mRNA methylation map has revealed the presence of thousands of methylated mRNAs [79]. A new molecular system formed by RBPs has been involved in adding (*writers*), removing (*erasers*) and recognizing (*readers*) chemical modifications present at the mRNA transcripts. The *A. thaliana* RNA Adenosine Methylase MTA (*writer*) has been described to be essential for plant survival as null *mta* mutants are embryo-lethal [80]. MTA is mainly expressed in dividing tissues, such as seeds upon development. Moreover, the expression of MTA under the control of the seed-specific ABI3 promoter rescues the lethality of null *mta* mutants, indicating its relevance during seed development [81].

### 3.2. Regulation of Seed Development through DNA Methylation

#### 3.2.1. DNA Methylation Basis

The chemical modification of DNA by the addition of a methyl group at the fifth position on the pyrimidine ring of cytosines is a stable and reversible epigenetic mark [82]. Centromeres and repeated sequences are heterochromatic regions, characterized by heavily methylated DNA [83]. DNA methylation in euchromatic regions and within genes has different effects on gene expression. While methylation within transcribed regions (the body of genes, mostly methyl-CG) is associated with constitutive expression levels [84], DNA methylation within promoters is associated with repression, i.e., gene silencing [85] and tissue-specific expression [83]. DNA methylation is a conserved epigenetic mechanism that, in plants, occurs at three different sequence contexts: the symmetrical CG and CHG, and the asymmetrical CHH, where H can be a cytosine, a thymidine or an adenine. Symmetrical methylation can be maintained during the cell cycle by DNA METHYLTRANSFERASE 1 (MET1) and CHROMOMETHYLASE 3 (CMT3), which catalyse DNA methylation on hemi-methylated DNA strands [86]. Asymmetrical methylation requires re-establishment after each DNA replication round by persistent de novo methylation by DOMAINS REARRANGED METHYLTRANSFERASE 2 (DRM2) at transposons and other repeated sequences in euchromatic chromosome arms. In this case, small RNAs cause transcriptional gene silencing by directing the addition of DNA methylation to specific DNA sequences by nucleotide homology mediated by ARGONAUTE (AGO) proteins, in a pathway called RNA-directed DNA methylation (RdDM) [87]. CHROMOMETHYLASE 2 (CMT2) catalyses CHH methylation at histone H1-containing heterochromatin, where DRM2 is inhibited [88]. Alternatively, sequences that have been methylated can become demethylated either passively or actively. Passive demethylation is due to subsequent rounds of DNA replication without the maintenance of the methylation pattern. Active demethylation is catalysed by DNA glycosylases —DEMETER (DME), REPRESSOR OF SILENCING1/DEMETER-LIKE 1 (ROS1), DEMETER-LIKE 2 (DML2) and DEMETER-LIKE 3 (DML3), in *A. thaliana* [89].

#### 3.2.2. DNA-Methylation and Seed Development

**Figure 2:**
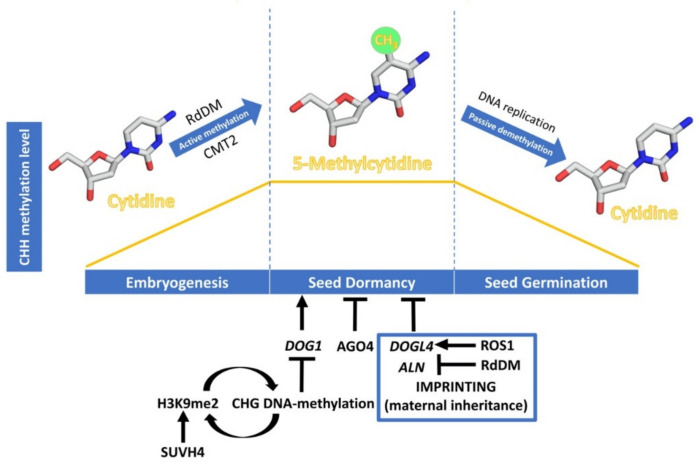
Dynamics of CHH DNA methylation (orange line) during seed development, and epigenetic regulation of seed dormancy. The DNA methylation process has been schematised with cytidine and 5-methylcytidine molecules designed using Chimera 1.14 [98]. Epigenetic regulation of seed dormancy has been schematised with arrows indicating a promotive effect and bars indicating a repressive effect. Regulation through genomic imprinting is indicated within the blue box.

The initial observations of cell nuclei, during *A. thaliana* seed maturation, have shown small size and very condensed chromatin, while the contrary happens during seed germination, suggesting a change in the balance between heterochromatin and euchromatin in the transition to germination [90]. Recent whole-genome bisulfite sequencing analysis supports these previous observations and shows that DNA methylation is dynamic during the process of seed development [91,92,93,94,95] (Figure 2). DNA methylation is known to play a critical role in seed development since mutations in MET1 and DME have been described to affect this developmental process [96,97]. Now, we know that during seed development there is a specific increase in global non-CG methylation, mainly due to changes in methyl-CHH and especially at transposable elements (TEs), both in *Glycine max* and *A. thaliana* [91,92,93,94,95]. This increase in global non-CG methylation occurs simultaneously throughout the seed, while CG and CHG methylations do not change significantly during seed development or in any specific part of it [91,92,94]. In mature embryos, CHH hypermethylation in TEs matches DNA hypomethylation in the endosperm. A role for RdDM in the establishment of this methylation is supported by the fact that 24-nt siRNAs derived from these regions are most abundant during late embryogenesis, coinciding with elevated expression of RdDM components [93]. Analysis of *drm1 drm2 cmt3* (*ddc*) and *drm1 drm2 cmt3 cmt2* (*ddcc*) mutants shows that CMT2 is also involved in this TE hypermethylation by the identification of over 100 de-repressed TEs which show different CHH methylation compared to that of randomly selected TEs [91]. This fact reinforces the idea that non-CG methylation and, specifically, methyl-CHH, may be an important mechanism to avoid TE activity during seed maturation [94]. Regarding DNA methylation within genes, it has been found that those genes that are transcribed are more likely to be differentially methylated during seed maturation than non-expressed genes [92]. The majority of these differentially methylated genes become transcriptionally down-regulated as seed maturation proceeds—DNA methylation levels increase—pointing to a specific role of DNA methylation in the repression of specific genes [92].

The expression levels of *DOG1* are regulated by various molecular mechanisms like alternative splicing, selective polyadenylation, and non-coding RNAs [18,99], but in this review we will highlight the consequences of *DOG1* H3K9 and DNA methylation in the regulation of seed dormancy. DNA methylation in the CHG context is perpetuated in *A. thaliana* by the SUVH family of histone H3 lysine 9 N-methyltransferases. Their SRA domain binds to methylated CHG cytosines, leading to H3K9me2 deposition. Conversely, sites of H3K9 methylation recruit DNA methyltransferases CMT3 and CMT2, thus forming a self-reinforcing loop of repressive epigenetic marks. The seeds of SUVH4 mutants (also known as *kyp*) show enhanced dormancy and up-regulation of *DOG1* and *ABI3* [100], supporting that histone H3K9me2 caused by KYP/SUVH4 induces their silencing through DNA methylation, negatively affecting seed dormancy. A possible role of the RdDM pathway in the silencing of seed dormancy genes has been suggested from studies in cereals. In barley, the *AGO4_9* gene *AGO1003* is expressed differentially in the embryos of dormant and non-dormant grains [101]. In wheat, *AGO802B*, a wheat ortholog of an *AGO4_9*, is expressed during grain development and its expression is significantly lower in preharvest sprouting-resistant varieties than in susceptible ones; implying that DNA methylation levels could be reduced in PHS susceptible varieties compared to resistant lines [102]. This result indicates that AGO4 is a negative regulator of dormancy, although it is not known whether specific coding genes are subjected to silencing through RdDM or which ones are. Recently, it has been demonstrated that mutants with loss or reduced function of the chromatin-remodelling factor PICKLE (PKL) show increased seed dormancy; PKL directly represses *DOG1* expression [103].

Genomic imprinting is an epigenetic mechanism that alters gene expression in a parent-of-origin manner. As a consequence, the expression of some genes depends on whether the allele was inherited from the maternal or the paternal parent [104]. Reciprocal crosses between *A. thaliana* accessions with increased seed dormancy—Cvi—and with reduced seed dormancy—C24—have suggested a maternal inheritance of seed dormancy levels. Thus, the two populations of F-1 hybrid seeds from these reciprocal crosses exhibit distinct levels of dormancy, which tend to phenocopy the maternal traits. In parallel, 57 dormancy-specific maternally expressed genes have been analysed [105], but the molecular mechanisms underpinning the regulation of these genes are yet to be elucidated. Recent studies have shown the importance of genomic imprinting, through DNA-methylation, in regulating the expression in the endosperm of two maternally expressed negative regulators of seed dormancy, DOG1-LIKE 4 (DOGL4) and ALLANTOINASE (ALN) [106,107]. In *A. thaliana*, active DNA demethylation depends on the activity of ROS1, which directly excises methyl-C from DNA [89]. Interestingly, *ros1* mutants are hypersensitive to ABA during early seedling development [108], showing that ROS1-driven DNA demethylation regulates seed dormancy, and the response to ABA by controlling the expression of DOGL4, a negative regulator of seed dormancy and the ABA response. ROS1 appears to antagonize the RdDM pathway, required for the imprinting that underlies the suppression of the paternal allele, by reducing the high level of DNA methylation of the paternal DOGL4 promoter, and consequently the gene repression. While both *dogl4* and *ros1* mutants show a decrease in germination rates and an increase in the response to ABA, overexpression of DOGL4 in *ros1* mutant background reverts the enhanced seed dormancy and ABA hypersensitivity phenotype, which shows that ROS1 regulation of seed dormancy is via DOGL4 [106].

DNA methylation also plays an important role in regulating the preferential maternal *ALN* expression in the seed endosperm. Higher CHH methylation levels in a transposable element present in the 5′ region of *ALN*, targeted by RdDM, correlate with lower expression of the gene. Paternal allele DNA methylation appears to be established during the gametogenesis in sperm cells in this gene [107]. During seed development, cold induces further CHH methylation in this region. This mechanism might allow seeds to store information about cold temperatures and optimize the germination timing. Upon germination, cold-induced DNA methylation is lost, allowing optimal gene expression in the next generation [107]. Further studies are required to understand how the RdDM machinery can discriminate paternal from maternal alleles and maintain the different levels of methylation after fertilization. It is suggested that the RdDM machinery might act preferentially at methylated DNA or that secondary imprinting such as histone modification might direct RdDM to the paternal allele [107].

During germination, although CG and CHG methylations remain stable, which indicates that MET1 and CMT3 are active in maintenance, there is a drop in CHH methylation levels. There is, however, no decrease in siRNAs for these hypomethylated loci nor in RdDM machinery components abundance, which points to demethylation causing the drop [91,95]. DNA demethylases are weakly or not expressed during germination, and demethylases mutants show no significant changes in DNA methylation when compared to wild type [91]. This suggests that passive demethylation may be behind the decrease in DNA methylation levels during the process of germination, although further studies are still needed to support this assertion. More than 12,000 differential methylated regions have been found in analyses upon *A. thaliana* seed germination, with a different degree of methylation in promoters of over 1800 differently expressed genes [95]. This implies that the dynamics of DNA methylation can regulate germination-related gene expression. Nevertheless, further studies on the specific set of genes regulated by changes in DNA methylation levels are required to understand the importance of this epigenetic regulation.

## 4. Concluding Remarks and Future Perspectives

Although the loss of seed dormancy through AR is a fact observed in a good number of seeds, the molecular mechanisms that govern the AR process are not entirely known. It is widely accepted that oxidation caused by ROS is the main mechanism leading the AR process. Oxidation can be a selective mechanism for degrading specific mRNAs related to proteins that inhibit germination or promote dormancy. The recruitment of mRNAs by RNA binding proteins (RBPs) that protect them during the storage period has been also proposed as a selective mechanism. These results indicate the existence of a specific mechanism that selects long-lived mRNAs essential for the progression of seed germination (Figure 1). It is also proposed that this selective mechanism is susceptible to environmental changes [34]. However, a more detailed molecular scenery is needed to establish the specific players (RBPs) controlling mRNA stability, and to know if other mRNA features are determining its stability upon seed dormancy. Apart from oxidation, other mRNA chemical modifications have been described, such as the methylation of adenosine (m^6^A) and cytosine (m^5^C) that could modulate transcript stability. In *A. thaliana*, the RNA Adenosine Methylase MTA has been found to be essential for embryo development [80]. New studies approaching this issue are needed to know if this interesting and emerging level to control expression could affect seed dormancy and AR.

DNA methylation is a broadly known epigenetic mechanism controlling gene expression. During *A. thaliana* embryogenesis, there is a global increase in CHH-context methylation, especially at TEs, where both RdDM and CMT2 pathways are involved. Global DNA methylation levels remain stable during seed dormancy and show a substantial loss during germination. This decrease in DNA methylation seems to be mainly due to passive demethylation through subsequent rounds of DNA replication without the maintenance of the methylation pattern. Interestingly, a self-reinforcing loop of repressive epigenetic marks seems to control *DOG1* expression during seed dormancy. The relevance of genomic imprinting, through DNA-methylation, in regulating the maternal expression of *DOG4* and *ALN*, negative regulators of seed dormancy, has been reported in the endosperm of *A. thaliana* seeds. However, the presence of specific DNA methylation marks associated with dormancy or germination transcriptomes remains to be elucidated.

The release of dormancy through AR is considered a major factor controlling soil seed germination in areas where the climate is generally warm and summer is dry, such as occurs in deserts and Mediterranean regions [109,110]. These territories occupy a significant part of the earth: deserts cover over 25% of the land areas, and the five Mediterranean-climate regions more than 2%, where the vascular plant flora comprises approx. 20% of the plant species in the world [111]. From an ecological point of view, the AR requisite avoids seed germinating when a high probability of seedling death exists, due to the elevated temperatures and low rainfalls during the summer [109]. Thus, the AR process can guarantee the species survival and ecosystem biodiversity, highlighting its ecological significance. The molecular mechanisms governing dormancy release through the AR process are not completely known, and the convergent pathways with the stratification process are still undiscovered. Moreover, most of the studies on AR have been carried out in *A. thaliana* ecotypes. This has paved the way to understand this fundamental process of seed physiology, but more knowledge in other species is required. In conclusion, new research about how ROS and nucleic acid modifications interact with other dormancy pathways (i.e., stratification) in *A. thaliana* and the other species would shed light to the uncompleted landscape of dormancy and AR. This will provide new tools for solving relevant agricultural problems such as pre-harvest sprouting.

## References

[1] Vicente-Carbajosa J., Carbonero P. (2005). Seed maturation: Developing an intrusive phase to accomplish a quiescent state. Int. J. Dev. Biol..

[2] Cadman C.S.C., Toorop P.E., Hilhorst H.W.M., Finch-Savage W.E. (2006). Gene expression profiles of Arabidopsis Cvi seeds during dormancy cycling indicate a common underlying dormancy control mechanism. Plant J..

[3] Holdsworth M.J., Bentsink L., Soppe W.J.J. (2008). Molecular networks regulating Arabidopsis seed maturation, after-ripening, dormancy and germination. New Phytol..

[4] Penfield S. (2017). Seed dormancy and germination. Curr. Biol..

[5] Gubler F., Millar A.A., Jacobsen J.V. (2005). Dormancy release, ABA and pre-harvest sprouting. Curr. Opin. Plant Biol..

[6] Finkelstein R., Reeves W., Ariizumi T., Steber C. (2008). Molecular aspects of seed dormancy. Annu. Rev. Plant Biol..

[7] Barrero J.M., Millar A.A., Griffiths J., Czechowski T., Scheible W.R., Udvardi M., Reid J.B., Ross J.J., Jacobsen J.V., Gubler F. (2010). Gene expression profiling identifies two regulatory genes controlling dormancy and ABA sensitivity in Arabidopsis seeds. Plant J..

[8] Abraham Z., Iglesias-Fernández R., Martínez M., Rubio-Somoza I., Díaz I., Carbonero P., Vicente-Carbajosa J. (2016). A developmental switch of gene expression in the barley seed mediated by HvVP1 (Viviparous-1) and HvGAMYB interactions. Plant Physiol..

[9] Shu K., Liu X., Xie Q., He Z. (2016). Two Faces of One Seed: Hormonal regulation of dormancy and germination. Mol. Plant.

[10] Carbonero P., Iglesias-Fernández R., Vicente-Carbajosa J. (2017). The AFL subfamily of B3 transcription factors: Evolution and function in angiosperm seeds. J. Exp. Bot..

[11] Nonogaki H. (2019). The long-standing paradox of seed dormancy unfolded?. Trends Plant Sci..

[12] Nakabayashi K., Bartsch M., Xiang Y., Miatton E., Pellengahr S., Yano R., Seo M., Soppe W.J.J. (2012). The time required for dormancy release in Arabidopsis is determined by DELAY OF GERMINATION1 protein levels in freshly harvested seeds. Plant Cell.

[13] Alonso-Blanco C., Bentsink L., Hanhart C.J., Blankestijn-de Vries H., Koornneef M. (2003). Analysis of natural allelic variation at seed dormancy loci of *Arabidopsis thaliana*. Genetics.

[14] Bentsink L., Jowett J., Hanhart C.J., Koornneef M. (2006). Cloning of DOG1, a quantitative trait locus controlling seed dormancy in Arabidopsis. Proc. Natl. Acad. Sci. USA.

[15] Née G., Xiang Y., Soppe W.J.J. (2017). The release of dormancy, a wake-up call for seeds to germinate. Curr. Opin. Plant Biol..

[16] Nishimura N., Tsuchiya W., Moresco J.J., Hayashi Y., Satoh K., Kaiwa N., Irisa T., Kinoshita T., Schroeder J.I., Yates J.R. (2018). Control of seed dormancy and germination by DOG1-AHG1 PP2C phosphatase complex via binding to heme. Nat. Commun..

[17] Nonogaki H. (2020). A repressor complex silencing ABA signaling in seeds?. J. Exp. Bot..

[18] Carrillo-Barral N., del Carmen Rodríguez-Gacio M., Matilla A.J. (2020). Delay of Germination-1 (DOG1): A key to understanding seed dormancy. Plants.

[19] Baskin J.M., Baskin C.C. (2004). A classification system for seed dormancy. Seed Sci. Res..

[20] Iglesias-Fernández R., Matilla A. (2009). After-ripening alters the gene expression pattern of oxidases involved in the ethylene and gibberellin pathways during early imbibition of Sisymbrium officinale L. seeds. J. Exp. Bot..

[21] Chahtane H., Kim W., Lopez-Molina L. (2017). Primary seed dormancy: A temporally multilayered riddle waiting to be unlocked. J. Exp. Bot..

[22] Buijs G., Vogelzang A., Nijveen H., Bentsink L. (2019). Dormancy cycling: Translation-related transcripts are the main difference between dormant and non-dormant seeds in the field. Plant J..

[23] Donohue K., Dorn L., Griffith C., Kim E.S., Aguilera A., Polisetty C.R., Schmitt J. (2005). The evolutionary ecology of seed germination of *Arabidopsis thaliana*: Variable natural selection on germination timing. Evolution.

[24] Schmuths H., BACHMANN K., WEBER W.E., HORRES R., HOFFMANN M.H. (2006). Effects of preconditioning and temperature during germination of 73 natural accessions of *Arabidopsis thaliana*. Ann. Bot..

[25] Nakabayashi K., Okamoto M., Koshiba T., Kamiya Y., Nambara E. (2005). Genome-wide profiling of stored mRNA in *Arabidopsis thaliana* seed germination: Epigenetic and genetic regulation of transcription in seed. Plant J..

[26] Carrera E., Holman T., Medhurst A., Dietrich D., Footitt S., Theodoulou F.L., Holdsworth M.J. (2008). Seed after-ripening is a discrete developmental pathway associated with specific gene networks in Arabidopsis. Plant J..

[27] Carrillo-Barral N., Matilla A.J., Iglesias-Fernández R., del Carmen Rodríguez-Gacio M. (2013). Nitrate-induced early transcriptional changes during imbibition in non-after-ripenedSisymbrium officinaleseeds. Physiol. Plant..

[28] Carrillo-Barral N., Matilla A.J., del Carmen Rodríguez-Gacio M., Iglesias-Fernández R. (2014). Nitrate affects sensu-stricto germination of after-ripened *Sisymbrium officinale* seeds by modifying expression of *SoNCED5*, *SoCYP707A2* and *SoGA3ox2* genes. Plant Sci..

[29] Dekkers B.J.W., Pearce S.P., van Bolderen-Veldkamp R.P.M., Holdsworth M.J., Bentsink L. (2016). Dormant and after-Ripened *Arabidopsis thaliana* seeds are distinguished by early transcriptional differences in the imbibed state. Front. Plant Sci..

[30] Bazin J., Langlade N., Vincourt P., Arribat S., Balzergue S., El-Maarouf-Bouteau H., Bailly C. (2011). Targeted mRNA oxidation regulates sunflower seed dormancy alleviation during dry after-ripening. Plant Cell.

[31] Bailly C. (2019). The signalling role of ROS in the regulation of seed germination and dormancy. Biochem. J..

[32] Finch-Savage W.E., Cadman C.S.C., Toorop P.E., Lynn J.R., Hilhorst H.W.M. (2007). Seed dormancy release in Arabidopsis Cvi by dry after-ripening, low temperature, nitrate and light shows common quantitative patterns of gene expression directed by environmentally specific sensing. Plant J..

[33] Sano N., Permana H., Kumada R., Shinozaki Y., Tanabata T., Yamada T., Hirasawa T., Kanekatsu M. (2012). Proteomic analysis of embryonic proteins synthesized from long-Lived mRNAs during germination of rice seeds. Plant Cell Physiol..

[34] Galland M., Huguet R., Arc E., Cueff G., Job D., Rajjou L. (2014). Dynamic proteomics emphasizes the importance of selective mRNA translation and protein turnover during Arabidopsis seed germination. Mol. Cell. Proteom..

[35] Sano N., Rajjou L., North H.M. (2020). Lost in translation: Physiological roles of stored mRNAs in seed germination. Plants.

[36] Xiao W., Custard R.D., Brown R.C., Lemmon B.E., Harada J.J., Goldberg R.B., Fischer R.L. (2006). DNA methylation is critical for Arabidopsis embroyogenesis and seed viability. Plant Cell.

[37] Jeevan Kumar S.P., Rajendra Prasad S., Banerjee R., Thammineni C. (2015). Seed birth to death: Dual functions of reactive oxygen species in seed physiology. Ann. Bot..

[38] Buijs G., Kodde J., Groot S.P.C., Bentsink L. (2018). Seed dormancy release accelerated by elevated partial pressure of oxygen is associated with DOG loci. J. Exp. Bot..

[39] Kurek K., Plitta-Michalak B., Ratajczak E. (2019). Reactive Oxygen species as potential drivers of the seed aging process. Plants.

[40] Oracz K., Bouteau H.E.-M., Farrant J.M., Cooper K., Belghazi M., Job C., Job D., Corbineau F., Bailly C. (2007). ROS production and protein oxidation as a novel mechanism for seed dormancy alleviation. Plant J..

[41] Müller K., Carstens A.C., Linkies A., Torres M.A., Leubner-Metzger G. (2009). The NADPH-oxidase AtrbohB plays a role in Arabidopsis seed after-ripening. New Phytol..

[42] Ebone L.A., Caverzan A., Chavarria G. (2019). Physiologic alterations in orthodox seeds due to deterioration processes. Plant Physiol. Biochem..

[43] De Giorgi J., Piskurewicz U., Loubery S., Utz-Pugin A., Bailly C., Mène-Saffrané L., Lopez-Molina L. (2015). An endosperm-associated cuticle is required for Arabidopsis seed viability, dormancy and early control of germination. PLOS Genet..

[44] Nakabayashi K., Bartsch M., Ding J., Soppe W.J.J. (2015). Seed dormancy in Arabidopsis requires self-binding ability of DOG1 Protein and the presence of multiple isoforms generated by alternative splicing. PLOS Genet..

[45] Liu Y., Ye N., Liu R., Chen M., Zhang J. (2010). H_2_O_2_ mediates the regulation of ABA catabolism and GA biosynthesis in Arabidopsis seed dormancy and germination. J. Exp. Bot..

[46] Barba-Espín G., Díaz-Vivancos P., Job D., Belghazi M., Job C., Hernández J.A. (2011). Understanding the role of H_2_O_2_ during pea seed germination: A combined proteomic and hormone profiling approach. Plant. Cell Environ..

[47] Yu Y., Zhen S., Wang S., Wang Y., Cao H., Zhang Y., Li J., Yan Y. (2016). Comparative transcriptome analysis of wheat embryo and endosperm responses to ABA and H_2_O_2_ stresses during seed germination. BMC Genom..

[48] Ishibashi Y., Aoki N., Kasa S., Sakamoto M., Kai K., Tomokiyo R., Watabe G., Yuasa T., Iwaya-Inoue M. (2017). The interrelationship between abscisic acid and reactive oxygen species plays a key role in barley seed dormancy and germination. Front. Plant Sci..

[49] Leymarie J., Vitkauskaité G., Hoang H.H., Gendreau E., Chazoule V., Meimoun P., Corbineau F., El-Maarouf-Bouteau H., Bailly C. (2012). Role of reactive oxygen species in the regulation of Arabidopsis seed dormancy. Plant Cell Physiol..

[50] Morscher F., Kranner I., Arc E., Bailly C., Roach T. (2015). Glutathione redox state, tocochromanols, fatty acids, antioxidant enzymes and protein carbonylation in sunflower seed embryos associated with after-ripening and ageing. Ann. Bot..

[51] Vigliocco A., Del Bel Z., Pérez-Chaca M.V., Molina A., Zirulnik F., Andrade A.M., Alemano S. (2019). Spatiotemporal variations in salicylic acid and hydrogen peroxide in sunflower seeds during transition from dormancy to germination. Physiol. Plant..

[52] Müller K., Linkies A., Vreeburg R.A.M., Fry S.C., Krieger-Liszkay A., Leubner-Metzger G. (2009). In vivo cell wall loosening by hydroxyl radicals during cress seed germination and elongation growth. Plant Physiol..

[53] Oracz K., Voegele A., Tarkowská D., Jacquemoud D., Turečková V., Urbanová T., Strnad M., Sliwinska E., Leubner-Metzger G. (2012). Myrigalone A inhibits *Lepidium sativum* seed germination by interference with gibberellin metabolism and apoplastic superoxide production required for embryo extension growth and endosperm rupture. Plant Cell Physiol..

[54] Waszczak C., Carmody M., Kangasjärvi J. (2018). Reactive Oxygen Species in plant signaling. Annu. Rev. Plant Biol..

[55] Oracz K., El-Maarouf-Bouteau H., Kranner I., Bogatek R., Corbineau F., Bailly C. (2009). The mechanisms involved in seed dormancy alleviation by hydrogen cyanide unravel the role of eactive Oxygen Species as key factors of cellular signaling during germination. Plant Physiol..

[56] Bahin E., Bailly C., Sotta B., Kranner I., Corbineau F., Leymarie J. (2011). Crosstalk between reactive oxygen species and hormonal signalling pathways regulates grain dormancy in barley. Plant Cell Environ..

[57] Kaur K., Zhawar V.K. (2016). Antioxidant potential of fresh and after-ripened dry embryos of two wheat cultivars contrasting in drought tolerance. Indian J. Plant Physiol..

[58] Farooq M.A., Niazi A.K., Akhtar J., Saifullah Farooq M., Souri Z., Karimi N., Rengel Z. (2019). Acquiring control: The evolution of ROS-Induced oxidative stress and redox signaling pathways in plant stress responses. Plant Physiol. Biochem..

[59] Serrato A.J., Crespo J.L., Florencio F.J., Cejudo F.J. (2001). Characterization of two thioredoxins h with predominant localization in the nucleus of aleurone and scutellum cells of germinating wheat seeds. Plant Mol. Biol..

[60] Ortiz-Espín A., Iglesias-Fernández R., Calderón A., Carbonero P., Sevilla F., Jiménez A. (2017). Mitochondrial *AtTrxo1* is transcriptionally regulated by AtbZIP9 and AtAZF2 and affects seed germination under saline conditions. J. Exp. Bot..

[61] Xu F., Tang J., Gao S., Cheng X., Du L., Chu C. (2019). Control of rice pre-harvest sprouting by glutaredoxin-mediated abscisic acid signaling. Plant J..

[62] Chmielowska-Bąk J., Arasimowicz-Jelonek M., Deckert J. (2019). In search of the mRNA modification landscape in plants. BMC Plant Biol..

[63] Simms C.L., Hudson B.H., Mosior J.W., Rangwala A.S., Zaher H.S. (2014). An active role for the ribosome in determining the fate of oxidized mRNA. Cell Rep..

[64] Taddei F., Hayakawa H., Bouton M.-F., Cirinesi A.-M., Matic I., Sekiguchi M., Radman M. (1997). Counteraction by MutT protein of transcriptional errors caused by oxidative damage. Science.

[65] Gao F., Rampitsch C., Chitnis V.R., Humphreys G.D., Jordan M.C., Ayele B.T. (2013). Integrated analysis of seed proteome and mRNA oxidation reveals distinct post-transcriptional features regulating dormancy in wheat (*Triticum aestivum* L.). Plant Biotechnol. J..

[66] Gao F., Jordan M.C., Ayele B.T. (2018). Microarray dataset of after-ripening induced mRNA oxidation in wheat seeds. Data Br..

[67] Nelson S.K., Ariizumi T., Steber C.M. (2017). Biology in the dry seed: Transcriptome changes associated with dry seed dormancy and dormancy loss in the arabidopsis GA-insensitive sleepy1-2 mutant. Front. Plant Sci..

[68] Cléry A., Blatter M., Allain F.H.-T. (2008). RNA recognition motifs: Boring? Not quite. Curr. Opin. Struct. Biol..

[69] Chai Q., Singh B., Peisker K., Metzendorf N., Ge X., Dasgupta S., Sanyal S. (2014). Organization of ribosomes and nucleoids in *Escherichia coli* cells during growth and in quiescence. J. Biol. Chem..

[70] Bai B., van der Horst S., Cordewener J.H.G., America T.A.H.P., Hanson J., Bentsink L. (2020). Seed-stored mRNAs that are specifically associated to monosomes are translationally regulated during germination. Plant Physiol..

[71] El-Maarouf-Bouteau H., Meimoun P., Job C., Job D., Bailly C. (2013). Role of protein and mRNA oxidation in seed dormancy and germination. Front. Plant Sci..

[72] Chen H., Chu P., Zhou Y., Li Y., Liu J., Ding Y., Tsang E.W.T., Jiang L., Wu K., Huang S. (2012). Overexpression of AtOGG1, a DNA glycosylase/AP lyase, enhances seed longevity and abiotic stress tolerance in Arabidopsis. J. Exp. Bot..

[73] Madugundu G.S., Cadet J., Wagner J.R. (2014). Hydroxyl-radical-induced oxidation of 5-methylcytosine in isolated and cellular DNA. Nucleic Acids Res..

[74] Michalak M., Barciszewska M.Z., Barciszewski J., Plitta B.P., Chmielarz P. (2013). Global Changes in DNA methylation in seeds and seedlings of *Pyrus communis* after Seed desiccation and storage. PLoS ONE.

[75] Michalak M., Plitta-Michalak B.P., Naskręt-Barciszewska M., Barciszewski J., Bujarska-Borkowska B., Chmielarz P. (2015). Global 5-methylcytosine alterations in DNA during ageing of *Quercus robur* seeds. Ann. Bot..

[76] Shen L., Liang Z., Wong C.E., Yu H. (2019). Messenger RNA modifications in plants. Trends Plant Sci..

[77] Cui X., Liang Z., Shen L., Zhang Q., Bao S., Geng Y., Zhang B., Leo V., Vardy L.A., Lu T. (2017). 5-Methylcytosine RNA methylation in *Arabidopsis thaliana*. Mol. Plant.

[78] Vandivier L.E., Campos R., Kuksa P.P., Silverman I.M., Wang L.-S., Gregory B.D. (2015). Chemical modifications mark alternatively spliced and uncapped messenger RNAs in Arabidopsis. Plant Cell.

[79] Fray R.G., Simpson G.G. (2015). The Arabidopsis epitranscriptome. Curr. Opin. Plant Biol..

[80] Zhong S., Li H., Bodi Z., Button J., Vespa L., Herzog M., Fray R.G. (2008). MTA Iis an Arabidopsis Messenger RNA adenosine methylase and interacts with a homolog of a sex-specific splicing factor. Plant Cell.

[81] Bodi Z., Zhong S., Mehra S., Song J., Graham N., Li H., May S., Fray R.G. (2012). Adenosine methylation in Arabidopsis mRNA is associated with the 3′ end and reduced levels cause developmental defects. Front. Plant Sci..

[82] Diez C.M., Roessler K., Gaut B.S. (2014). Epigenetics and plant genome evolution. Curr. Opin. Plant Biol..

[83] Zhang X., Yazaki J., Sundaresan A., Cokus S., Chan S.W.L., Chen H., Henderson I.R., Shinn P., Pellegrini M., Jacobsen S.E. (2006). Genome-wide high-resolution mapping and functional analysis of DNA methylation in Arabidopsis. Cell.

[84] Bewick A.J., Schmitz R.J. (2017). Gene body DNA methylation in plants. Curr. Opin. Plant Biol..

[85] Zilberman D., Gehring M., Tran R.K., Ballinger T., Henikoff S. (2007). Genome-wide analysis of *Arabidopsis thaliana* DNA methylation uncovers an interdependence between methylation and transcription. Nat. Genet..

[86] Law J.A., Jacobsen S.E. (2010). Establishing, maintaining and modifying DNA methylation patterns in plants and animals. Nat. Rev. Genet..

[87] Matzke M.A., Kanno T., Matzke A.J.M. (2015). RNA-Directed DNA methylation: The evolution of a complex epigenetic pathway in flowering plants. Annu. Rev. Plant Biol..

[88] Zhang H., Lang Z., Zhu J.-K. (2018). Dynamics and function of DNA methylation in plants. Nat. Rev. Mol. Cell Biol..

[89] Parrilla-Doblas J.T., Roldán-Arjona T., Ariza R.R., Córdoba-Cañero D. (2019). Active DNA Demethylation in plants. Int. J. Mol. Sci..

[90] van Zanten M., Koini M.A., Geyer R., Liu Y., Brambilla V., Bartels D., Koornneef M., Fransz P., Soppe W.J.J. (2011). Seed maturation in *Arabidopsis thaliana* is characterized by nuclear size reduction and increased chromatin condensation. Proc. Natl. Acad. Sci. USA.

[91] Kawakatsu T., Nery J.R., Castanon R., Ecker J.R. (2017). Dynamic DNA methylation reconfiguration during seed development and germination. Genome Biol..

[92] An Y.C., Goettel W., Han Q., Bartels A., Liu Z., Xiao W. (2017). Dynamic changes of genome-wide DNA methylation during soybean seed development. Sci. Rep..

[93] Bouyer D., Kramdi A., Kassam M., Heese M., Schnittger A., Roudier F., Colot V. (2017). DNA methylation dynamics during early plant life. Genome Biol..

[94] Lin J.-Y., Le B.H., Chen M., Henry K.F., Hur J., Hsieh T.-F., Chen P.-Y., Pelletier J.M., Pellegrini M., Fischer R.L. (2017). Similarity between soybean and Arabidopsis seed methylomes and loss of non-CG methylation does not affect seed development. Proc. Natl. Acad. Sci. USA.

[95] Narsai R., Gouil Q., Secco D., Srivastava A., Karpievitch Y.V., Liew L.C., Lister R., Lewsey M.G., Whelan J. (2017). Extensive transcriptomic and epigenomic remodelling occurs during *Arabidopsis thaliana* germination. Genome Biol..

[96] Choi Y., Gehring M., Johnson L., Hannon M., Harada J.J., Goldberg R.B., Jacobsen S.E., Fischer R.L. (2002). DEMETER, a DNA glycosylase domain protein, is required for endosperm gene imprinting and seed viability in Arabidopsis. Cell.

[97] Xiao W., Brown R.C., Lemmon B.E., Harada J.J., Goldberg R.B., Fischer R.L. (2006). Regulation of seed size by hypomethylation of maternal and paternal genomes. Plant Physiol..

[98] Pettersen E.F., Goddard T.D., Huang C.C., Couch G.S., Greenblatt D.M., Meng E.C., Ferrin T.E. (2004). UCSF Chimera—A visualization system for exploratory research and analysis. J. Comput. Chem..

[99] Li P., Ni H., Ying S., Wei J., Hu X. (2020). Teaching an old dog a new trick: Multifaceted strategies to control primary seed germination by DELAY OF GERMINATION 1 (DOG1). Phyton (B. Aires)..

[100] Zheng J., Chen F., Wang Z., Cao H., Li X., Deng X., Soppe W.J.J., Li Y., Liu Y. (2012). A novel role for histone methyltransferase KYP/SUVH4 in the control of Arabidopsis primary seed dormancy. New Phytol..

[101] Singh M., Singh J. (2012). Seed development-related expression of ARGONAUTE4_9 class of genes in barley: Possible role in seed dormancy. Euphytica.

[102] Singh M., Singh S., Randhawa H., Singh J. (2013). Polymorphic homoeolog of key gene of RdDM pathway, ARGONAUTE4_9 class is associated with pre-harvest sprouting in Wheat (*Triticum aestivum* L.). PLoS ONE.

[103] Zha P., Liu S., Li Y., Ma T., Yang L., Jing Y., Lin R. (2020). The evening complex and the chromatin-remodeling factor PICKLE coordinately control seed dormancy by directly repressing *DOG1* in Arabidopsis. Plant Commun..

[104] Köhler C., Wolff P., Spillane C. (2012). Epigenetic mechanisms underlying genomic imprinting in plants. Annu. Rev. Plant Biol..

[105] Piskurewicz U., Iwasaki M., Susaki D., Megies C., Kinoshita T., Lopez-Molina L. (2016). Dormancy-specific imprinting underlies maternal inheritance of seed dormancy in *Arabidopsis thaliana*. eLife.

[106] Zhu H., Xie W., Xu D., Miki D., Tang K., Huang C.-F., Zhu J.-K. (2018). DNA demethylase ROS1 negatively regulates the imprinting of *DOGL4* and seed dormancy in *Arabidopsis thaliana*. Proc. Natl. Acad. Sci. USA.

[107] Iwasaki M., Hyvärinen L., Piskurewicz U., Lopez-Molina L. (2019). Non-canonical RNA-directed DNA methylation participates in maternal and environmental control of seed dormancy. eLife.

[108] Kim J.-S., Lim J.Y., Shin H., Kim B.-G., Yoo S.-D., Kim W.T., Huh J.H. (2019). ROS1-dependent DNA demethylation is required for ABA-inducible NIC3 expression. Plant Physiol..

[109] Iglesias-Fernández R., Del Carmen Rodrguez-Gacio M., Matilla A.J. (2011). Progress in research on dry afterripening. Seed Sci. Res..

[110] Baskin C.C., Baskin J.M. (2014). Seeds: Ecology, Biogeography, and, Evolution of Dormancy and Germination.

[111] Klausmeyer K.R., Shaw M.R. (2009). Climate change, habitat loss, protected areas and the climate adaptation potential of species in mediterranean ecosystems worldwide. PLoS ONE.

